# Identification and Characterization of PLATZ Transcription Factors in Wheat

**DOI:** 10.3390/ijms21238934

**Published:** 2020-11-25

**Authors:** Yuxin Fu, Mengping Cheng, Maolian Li, Xiaojiang Guo, Yongrui Wu, Jirui Wang

**Affiliations:** 1 Triticeae Research Institute, Sichuan Agricultural University, Chengdu 611130, China; yuxinfu_anna@163.com (Y.F.); 71250@sicau.edu.cn (M.C.); 71296@sicau.edu.cn (M.L.); xiaojiang9945@gmail.com (X.G.); 2 National Key Laboratory of Plant Molecular Genetics, CAS Center for Excellence in Molecular Plant Sciences, Institute of Plant Physiology and Ecology, Shanghai Institutes for Biological Sciences, Chinese Academy of Sciences, Shanghai 200032, China; yrwu@sibs.ac.cn; 3 Key Laboratory for Crop Genetic Resources and Improvement in Southwest China, Sichuan Agricultural University, Ministry of Education, Chengdu 611130, China; 4 State Key Laboratory of Crop Gene Exploration and Use in Southwest China, Sichuan Agricultural University, Chengdu 611130, China

**Keywords:** wheat, transcription factor, PLATZ, seed-specific expression, duplication

## Abstract

The PLATZ (plant AT-rich protein and zinc-binding protein) transcription factor family is a class of plant-specific zinc-dependent DNA-binding proteins. PLATZ has essential roles in seed endosperm development, as well as promoting cell proliferation duration in the earlier stages of the crops. In the present study, 62 *TaPLATZ* genes were identified from the wheat genome, and they were unequally distributed on 15 chromosomes. According to the phylogenetic analysis, 62 *TaPLATZ* genes were classified into six groups, including two groups that were unique in wheat. Members in the same groups shared similar exon-intron structures. The polyploidization, together with genome duplication of wheat, plays a crucial role in the expansion of the *TaPLATZs* family. Transcriptome data indicated a distinct divergence expression pattern of *TaPLATZ* genes that could be clustered into four modules. The *TaPLATZs* in Module b possessed a seed-specific expression pattern and displayed obvious high expression in the earlier development stage of seeds. Subcellular localization data of *TaPLATZs* suggesting that they likely perform a function as a conventional transcription factor. This study provides insight into understanding the structure divergence, evolutionary features, expression profiles, and potential function of PLATZ in wheat.

## 1. Introduction

Transcription regulation of genes in response to developmental and environmental changes, mediated by the DNA-binding transcription factors (TFs), is an important regulatory mechanism in plants [1]. Transcription factors bind to the cis-acting elements upstream of gene promoters to activate or repress gene expression.

During the evolution of transcription factor families, the TFs between plants and animals or yeast do not always correspond. In Arabidopsis, over 5% of its genome is devoted to encoding more than 1500 transcription factors, approximately 45% of which are specific to plants [2]. Some classes of transcription factors appear to have specifically evolved in plants, such as the WRKY, NAC, and AP2/EREBP families, which strictly integrate gene regulatory networks in plant growth processes, including metabolism, hormone signaling, pathogen defense and senescence [3,4,5].

The PLATZ (plant AT-rich sequence and zinc-binding) TF family is a class of plant-specific zinc-dependent DNA-binding proteins whose first member PLATZ1 was isolated in pea. The pea PLATZ1 acts as a transcriptional repressor by nonspecifically binding to A/T-rich sequences [6]. Multiple sequence alignments show that PLATZ proteins possess two highly conserved zinc finger motifs comprising several cysteine and histidine residues, with C-x_2_-H-x_10_-C-x_2_-C-x(_4–5_)-C-x_2_-C-x(_3–7_)-H-x_2_-H at N-terminal region and C-x_2_-C-x(_10–11_)-C-x_3_-C [6], both of which have been described as being necessary for zinc-dependent DNA binding.

PLATZ proteins participate in environmental stress responses. The transcription levels of *GmPLATZ1*(*Glycine max PLATZ1*) were upregulated by ABA and drought stress in soybean. Germination in *GmPLATZ1*-overexpression transgenic Arabidopsis was retarded in response to ABA and osmotic stress [7].

The biological functions of PLATZ proteins were not well identified until the maize *Fl3* gene was cloned from a classic endosperm semi-dominant mutant [8]. *Fl3* (*ZmPLATZ12*) is specifically expressed in maize endosperm starchy cells, encoding a PLATZ protein, Fl3, that is required for transcription of transfer RNAs (tRNAs) and 5S ribosomal RNA (5S rRNA), through interaction with the RNA polymerase III (RNAPIII) subunits RPC53 and TFC1, which participate in the development of endosperm and filling of storage materials in maize seeds [3].

PLATZ proteins are involved in leaf growth and senescence. *ORESARA15* (*ORE 15*) encodes a PLATZ TF from Arabidopsis, promotes leaf growth by enhancing the rate and duration of cell proliferation in the leaf earlier development stage, and functions as a negative regulator of leaf senescence in the later stage by modulating the GRF/GIF regulatory pathway [9].

The *PLATZ* gene LOC_Os06g45540 from rice was mapped as a major QTL locus (*GL6)*, identified from flanking sequence analysis of Japonica T-DNA insertion mutant *sg6* almost simultaneously, which function in regulating grain sizes. *GL6* determines grain length and spikelet number by affecting cell proliferation through gene expression regulation via the RNA polymerase III transcription machinery [10]. *SG6* determines grain size by interacting with the core cell cycle machinery DP protein and regulating spikelet hull cell division [11].

PLATZ family members have been identified in several plant species, including 13 genes in the *Arabidopsis* genome, eight genes in *Glycine max*, nine genes in *Gossypium hirsutum*, 15 genes in *Oryza sativa*, and 17 genes in *Zea mays*. The PLATZ proteins from rice, maize, and *Arabidopsis* were clustered into five subfamilies based on their amino acid structures [12]. However, the characterization and function of PLATZ in wheat have not yet been elucidated.

Wheat (*Triticum aestivum* L., BBAADD) is a staple food crop for more than one-third of the global human population and provides approximately 20% of the calories consumed by humans globally. Wheat is an allohexaploid species originated from two hybridizations: first between *Triticum urartu* (AA) and *Aegilops speltoides* (SS), and second between allotetraploid wild emmer wheat [*Triticum turgidum* ssp., BBAA] and *Aegilops tauschii* (DD) [13,14,15]. Recently, high-quality genome sequencing and assembly have been applied to wheat [16,17]. The genome sequences serve as references for underpinning genome scans for phenotypic associations and gene cloning from wheat and their wild progenitors for the accelerated development of improved wheat varieties [18,19]. The availability of these reference sequences allows the identification of genes within genetic intervals [20].

Recent advances in wheat genomics have presented an opportunity to identify wheat PLATZ family genes in hexaploid wheat. Identification and functional characterization of *PLATZs* in wheat will provide an avenue for exploring and verifying the conserved and specific mechanism of PLATZ members in plant development. Here, we characterized wheat *PLATZs* with respect to the following aspects: (1) genome-wide identification of PLATZ genes in wheat; (2) analysis of conserved motifs and cis-elements of PLATZ genes; (3) identification of the chromosomal distribution and the gene duplications; (4) phylogenetic and evolutionary relationship of *PLATZ* in wheat associate with rice and maize; (5) elucidation of expression profiles of *PLATZ* genes; and (6) subcellular localization of *PLATZ* genes. The results provide conditions for further study of *PLATZ* genes concerning flower and fruit growth in wheat.

## 2. Results

### 2.1. Identification of the PLATZ Genes in Wheat

According to the Hidden Markov Model (HMM) profile (PF04640), a total of 50 candidate sequences were found in the IWGSC v1.1 high-confidence (HC) and low-confidence (LC) peptide database using the HMM search program. This dataset was simplified by keeping the first splice variant from each transcript for further analyses. An additional 13 sequences were identified using a BLAST search in the unannotated IWGSC v2.0 genomic database. Subsequently, the PLATZ domain in the identified sequences was confirmed in the Pfam database, SMART database, and NCBI CDD program. By getting rid of sequences with incomplete PLATZ domains, 62 sequences were identified as putative *PLATZ* family members for further analysis (Table S1). The number of *PLATZs* in wheat is approximately three times greater than that of Arabidopsis (13 *AtPLATZs*), maize (17 *ZmPLATZs*), and rice (15 *OsPLATZs*) [12,21]. These *TaPLATZ*s have 145 to 275 amino acids, with an average of 221 amino acids. The molecular weights of TaPLATZ were between 16.67 kDa and 29.76 kDa. The predicted pI values of TaPLATZ ranged from 5.21 to 9.68. The 62 identified *PLATZ* genes contain the conserved cysteine and histidine residues enrichment regions: C-x2-H-x(11–12)-C-x_2_-C-x(_4–8_)-C-x_2_-C-x(_3–4_)-H-x_2_-H and C-x_2_-C-x(_10–11_)-C-x_3_-C. The N-domain and C-domain conserved structure of PLATZ proteins are crucial for their zinc-binding ability [6].

### 2.2. Phylogenetic Tree and Conserved Motif Characterization of TaPLATZ Genes

**Figure 1:**
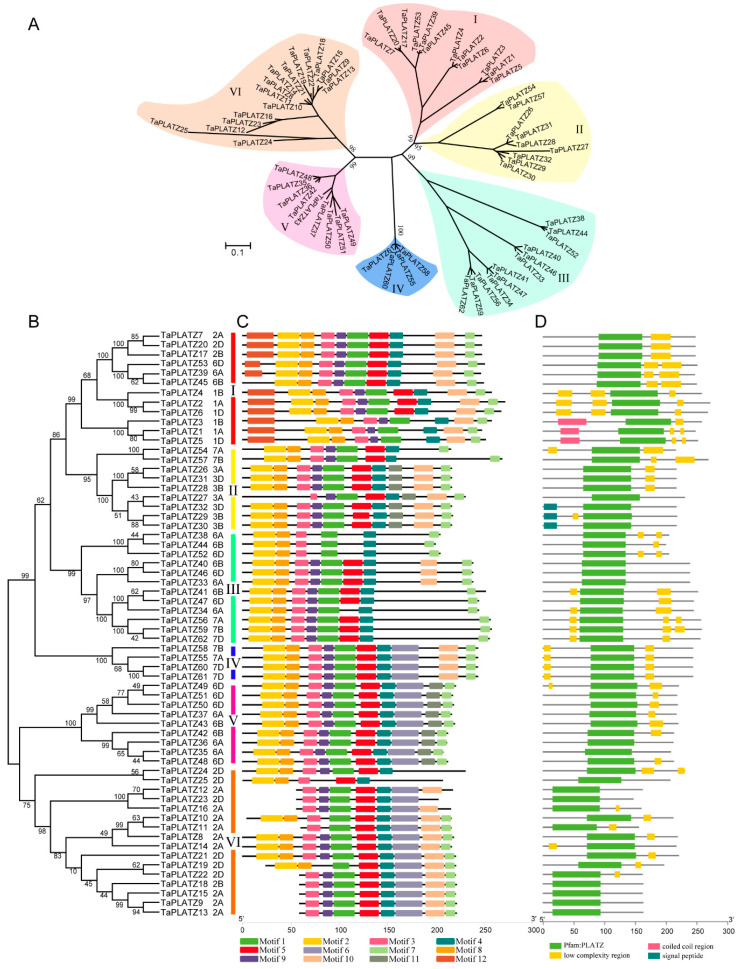
Phylogenetic relationship, motif structure, and conservation analysis of *TaPLATZ* genes. (**A**) An unrooted phylogenetic tree of *TaPLATZ* genes. Percent of bootstrap values are given for the main branches and support the classification in subfamilies; (**B**) The neighbor-joining tree of TaPLATZ proteins. I-VI: *TaPLATZs* were divided into six groups and are represented using different colors; (**C**) MEME motif structure shows the distinct divergence between groups; (**D**) Batch-smart analysis of PLATZ domain distribution of TaPLATZ proteins.

Multiple sequence alignment data of 62 *TaPLATZ* genes were used to construct a neighbor-joining phylogenetic tree and further explore the similarity and diversity of motif compositions. *TaPLATZ*s were classified into six groups (Groups I-VI) based on the topological structure of the phylogenetic tree (Figure 1A,B). Group VI was the largest one, containing 16 *TaPLATZ*s. Both Groups I and III had 12 *TaPLATZs*, followed by Group II and V with 9 *TaPLATZ*s. In contrast, Group IV contained the least, with four *TaPLATZs* (Table S5).

A neighbor-joining phylogenetic tree was carried out based on *PLATZ*s from wheat associated with maize, and rice (Figure 1A). In most groups, gene phylogeny followed species phylogeny [22]. Only wheat *PLATZs* could be found in groups V and VI. More members of wheat *PLATZs* could be found than that from maize and rice in groups I, III, and IV. Some *TaPLATZ* genes were orthologs to *ZmPLATZ* and *OsPLATZ* with 100% bootstrap value, such as *TaPLATZ33*, *TaPLATZ40*, *TaPLATZ46* orthologs to *ZmPLATZ9* and LOC_Os02g09070 (Group III), revealing the sequences conservation during species evolution.

A total of 12 motifs, named motif-1 to motif-12, were detected using the MEME online program (Table S4). The highly conserved motif distribution in each group ensures the classification of genes accurately and regulation of downstream genes precisely. However, these motifs showed distinct divergence among six groups (Figure 1C). Motif-2 and motif-8 matched to the conservation cysteine and histidine residues in the N-terminal of PLATZ protein, which was found in all groups. Motif-5 and motif-4 had the conservation cysteine residues in the C-terminal of PLATZs that were detected in all groups. Motif-7 represents the termination region of PLATZs in all groups, indicating the integrity of most PLATZs. Motif-10 was detected in all groups except Group V. Motif-6 was only detected in Group IV, Group V, and Group VI; and motif-11 was specific to Group II and V. Motif-12, located in the starting position of translation, was only observed in Group I. All *TaPLATZ*s contained PLATZ-conserved domains in the central region (Figure 1D), providing the ability for zinc-dependent DNA binding.

### 2.3. Collinearity Analysis and Gene Duplication of TaPLATZs

**Figure 2:**
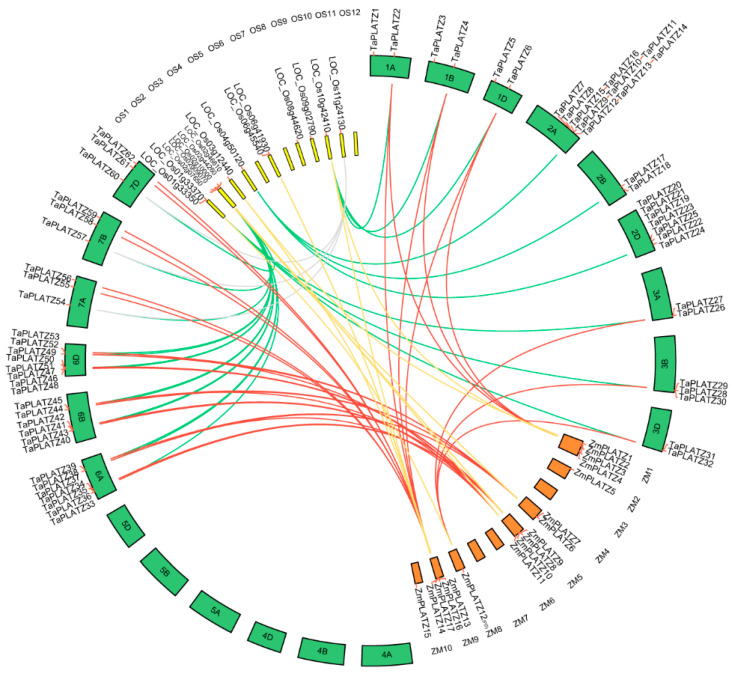
Collinear relationship analysis between orthologous *PLATZ* genes in wheat, rice, and maize. The circle atlas represents the orthologous pair position and collinearity. The red lines represent the orthologous *PLATZ* genes between wheat and maize. The green lines denote the orthologous *PLAYZ* genes in wheat and rice. The yellow lines represent the orthologous genes between maize and rice. Grey lines highlight the second orthologous pair of *TaPLATZ54*, *TaPLATZ57*, *TaPLATZ60* with rice. 1A–7D represent the twenty-one chromosomes of the wheat; OS1-OS12 represent the twelve chromosomes of rice; ZM1-ZM10 represent the ten chromosomes of maize.

Collinear relationships between 62 *TaPLATZ* genes, 15 *OsPLATZ* genes, and 17 *ZmPLATZ* genes were analyzed for a better understanding of *PLATZ* genes evolution (Table S2, Figure 2). A total of thirty *TaPLATZ* genes showed collinear relationships with those in maize and rice, indicating that these orthologous pairs may already exist before the ancestral divergence. In addition, some *TaPLATZ* genes were associated with two orthologous genes located on different chromosomes, such as collinear gene pairs (*TaPLATZ54*, *TaPLATZ57*, *TaPLATZ60*, LOC_Os08g44620, and LOC_Os11g24130). Some collinear gene pairs were detected only in wheat and rice, such as *TaPLATZ7*, *TaPLATZ17*, *TaPLATZ20*.

In wheat, all 62 *TaPLATZs* were found to be unequally distributed on fifteen chromosomes, except for chromosomes 4A, 4B, 4D, 5A, 5B, and 5D. Most of the *TaPLATZs* (40/62) were located on chromosomes 2A, 2B, 2D, 6A, 6B, and 6D. Meanwhile, chromosomes 1A, 1B, and 1D contained only two *TaPLATZs*, respectively. It is indicated that duplication events tended to occur in chromosomes 2 and 6 during the evolution of gene families, which may be associated with gene functions. *OsPLATZs* were distributed unevenly among the nine rice chromosomes, as well. There were no *PLATZs* on chromosomes 5, 7, and 12. In addition, *ZmPLATZ* also exhibited uneven distribution. None of the *PLATZs* could be found on chromosomes 3, 6, 7, and 10 of maize.

**Figure 3:**
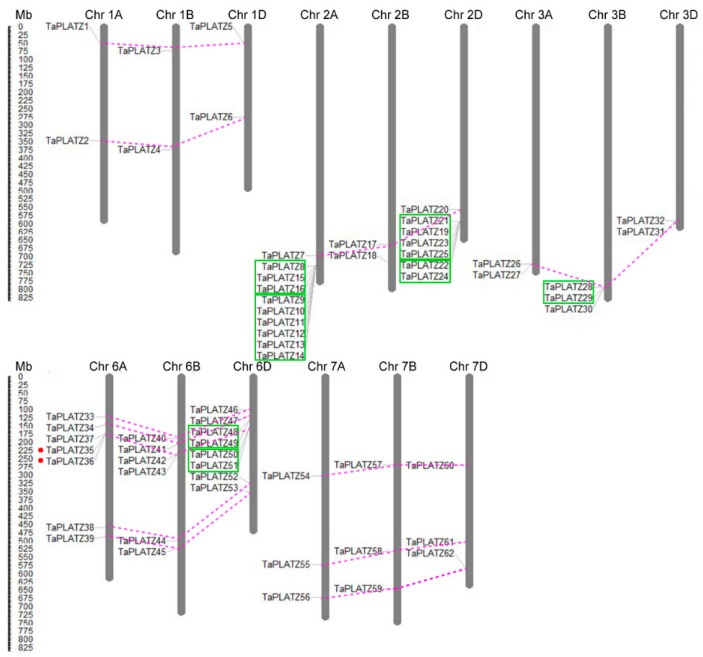
Distribution and duplication of *TaPLATZ* genes in *Triticum aestivum* chromosomes. The chromosome numbers are shown at the top of each bar. The length of chromosomes was their relative extent. The scale on the left is in megabases (Mb). Putative *TaPLATZ* homologous gene pairs were ligated with violet dotted lines. The tandem duplicated genes were marked with green boxes. Segmental duplicated genes were marked by red dots.

Furthermore, the physical locations of the *TaPLATZ* genes were mapped on the corresponding chromosomes for a better understanding of the duplication events (Figure 3). During the process of evolution, gene duplication is essential for the generation of novel biological functions and the expansion of the gene family [23]. In wheat, most *TaPLATZs* had the corresponding homoeologous on the A, B, and D sub-genomes. Forty-six *TaPLATZ* genes exhibited a homology of 1:1:1 on the three sub-genomes with high identity, and can be referred to as triplets, indicating that wheat polyploidization was the main reason the expansion of the wheat PLATZ family. Apart from triplets, some genes contain several homologues due to complex gene-duplication events during wheat evolution.

Duplication analysis revealed that obvious tandem duplication genes were found universally on chromosome 2A, 2D, 3B, and 6D (Figure 3) according to the criteria used in the analysis. Twenty-one out of 62 (33.9%) *TaPLATZ* genes were tandem duplicated, based on the similarity of their sequences and the proximity of position on the chromosome (Table S6). In addition, the *TaPLATZ* family has merely two segmental duplication genes (*TaPLA*TZ*35* and *TaPLAT*Z*36*), indicating that tandem duplication contributed more to the expansion of *TaPLATZ* family. Moreover, the majority of tandem duplicates were observed on subtelomeric distal regions of chromosomes result in more duplication events. On the other hand, genes located on the centromere proximal region of the chromosomes led to lower exchange frequency, such as *TaPLATZs* on chromosome 1A, 1B, and 1D.

### 2.4. Variety of Cis-Acting Elements in Promoter Regions of TaPLATZs

**Figure 4:**
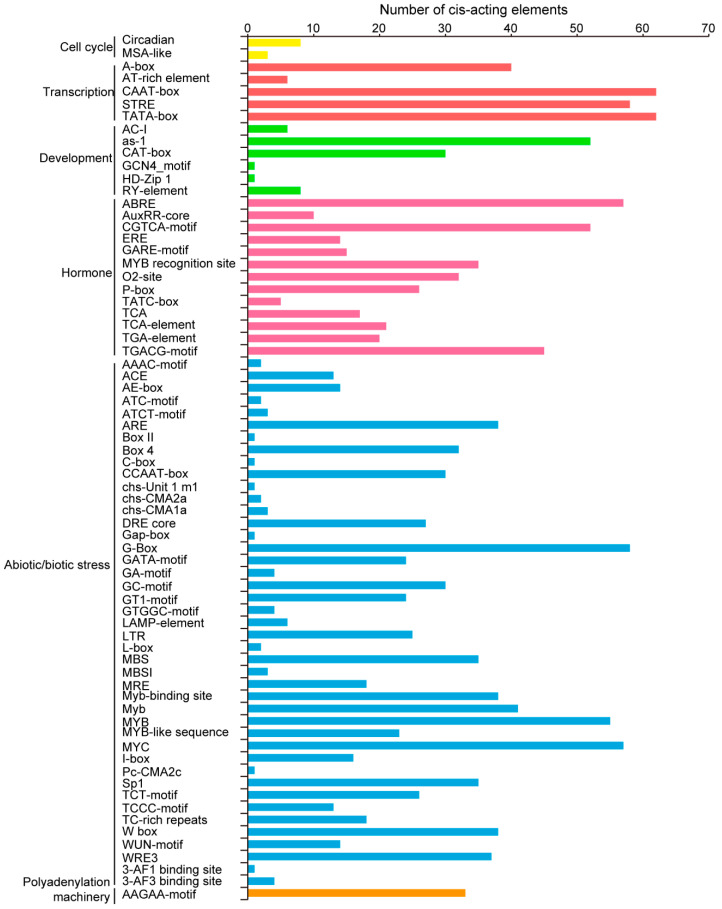
The number of cis-acting elements contained in the promoters of *TaPLATZ* genes. The cis-acting element was identified with the online PlantCARE program using the 2k upstream from the transcription start site of *TaPLATZ* genes. The graph was generated based on the presence of cis-acting elements related to different conditions (*x*-axis) in 62 *TaPLATZs* (*y*-axis). The number of cis-acting elements involved in different regulatory pathways. Yellow columns refer to cell cycle, red columns represent transcription, green columns represent of the development process, blue columns refer to abiotic and biotic stress, orange columns represent the polyadenylation machinery.

Studies of promoters that largely regulate gene expression at the transcriptional level are crucial for improving our basic understanding of gene regulation [24]. The 2.0-kb upstream sequences of translational initiation sites of *TaPLATZs* were used to predict cis-acting elements using the online database PlantCARE(Figure 4). Various cis-acting elements were found in the promoter of 62 *PLATZ* genes. Light responsive elements, including G-box (58/62), Sp1 (35/62), and Box 4 (32/62), were abundant in the 62 *TaPLATZ* genes. Drought-inducibility element MBS (35/62) accounted for a large part of the *TaPLATZ* genes, indicating the possibility of binding and regulation by the MYB transcription factor. Hormone responsive elements related to the response of gibberellin, salicylic acid (SA), abscisic acid (ABA), and methyl jasmonic acid (MEJA); as well as various abiotic stresses, such as ABRE (57/62), CGTCA-motif (52/62), and TGACG-motif (45/62), were identified in *TaPLATZ* genes.

The promoter-related elements CAAT-box and TATA-box were found in the promoter regions of all the 62 *TaPLATZ* genes, illustrating that most of the family members have transcriptional activity. Beyond that, eight *TaPLATZ* genes contained RY-element on promoters, such as *TaPLATZ54*, *TaPLATZ58*, and *TaPLATZ61*, giving rise to the possibility of regulating gene expression during late embryogenesis and seed development stage [25].

### 2.5. Expression Patterns of TaPLATZs

**Figure 5:**
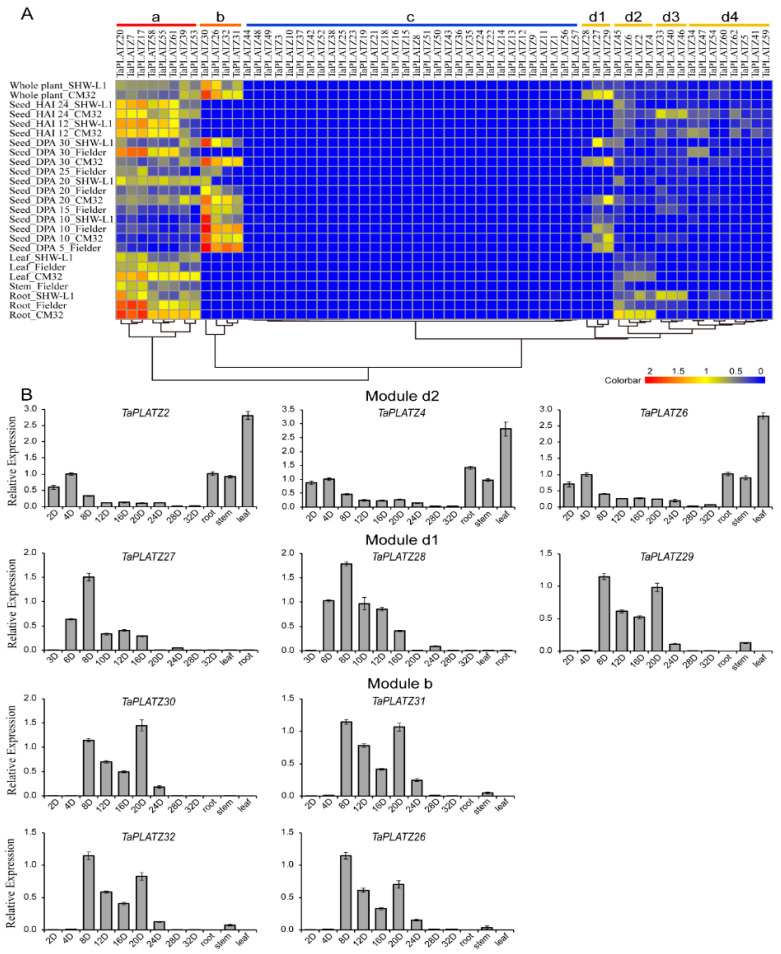
The expression profiles of *TaPLATZ*s in wheat. (**A**) Heat map of *TaPLATZ* genes expression in different tissues and developmental stages. Colorbar represents the expression abundance of RNA-seq data. CM32: Chuanmai 32; HAI 12: seeds after 12 h of imbibition; HAI 24: seeds after 24 h of imbibition; DPA: days post-anthesis. (**B**) Real-time PCR data of ten selected *TaPLATZs* from Modules b, d1, and d2 in different developmental stages of seeds, root, stem, and leaves.

The expression patterns of 62 *TaPLATZs* were characterized using the transcriptomes (RNA-seq) data at different growth stages or in different tissues of wheat (Figure 5A). *TaPLATZs* were clustered into four main modules (Module a to d) in the heat map according to expression abundance. Module d was further divided into four submodules (Module d1 to d4), respectively. Eight *TaPLATZs* in Module a were highly expressed in roots, stems, leaves, developmental seeds at later stages, which refer to days-post-anthesis (DPA 20, DPA 25, and DPA 30) and germinating seeds after 12 and 24 h of imbibition (HAI 12, HAI 24). Genes with seed-specific expression patterns were clustered in Module b. Three genes’ (*TaPLATZ26*, *TaPLATZ31*, *TaPLATZ32*) high expression levels remained in DPA 5 and DPA 10 seeds, which decreased gradually in DPA 20 and DPA 25. Additionally, the expression level of *TaPLATZ30* remained high in all of the developmental stages of the seeds. In Module d1, all genes (*TaPLATZ27*, *TaPLAT*Z*28*, and *TaPLAT*Z*29*) presented seed-specific expression patterns, but maintained a low level of expression. In Module d2, three genes (*TaPLATZ6*, *TaPLAT*Z2, and *TaPLATZ4*) were exclusively expressed in roots and leaves. Another three genes clustered in Module d3 (*TaPLATZ33*, *TaPLATZ40*, and *TaPLATZ46*) had an obvious high expression level in roots of SHW-L1 and seeds at HAI 12 and HAI 24 of Chuanmai 32. Two genes (*TaPLATZ34*, *TaPLATZ47*) belonging to Module d4 were only expressed in DPA 30 of Fielder, and seeds at HAI 12, and HAI 24 of Chuanmai 32. In Module c, low expression levels were only detected in a few genes. It is worthy of noting that most genes clustered in the same subfamily of the phylogenetic tree possessed corresponding expression patterns. In other words, homoeologous genes possessed similar expression patterns and further ensured the functional stability in the process of evolution.

Ten *TaPLATZs* were selected from Module b and Module d1 and d2 for real-time PCR, and their expression patterns were further determined in leaves, roots, stems, and different developmental stages of seeds (Figure 5B). The qPCR results of all the selected genes were significantly correlated with the RNA-seq data. The expression levels of three genes (*TaPLATZ2*, *TaPLATZ4*, *TaPLATZ6*) from Module d2 were relatively high in roots and leaves. Three genes (*TaPLATZ27*, *TaPLATZ28*, *TaPLATZ29*) clustered in Module d1, and four genes (*TaPLATZ30*, *TaPLATZ26*, *TaPLATZ32*, *TaPLATZ31*) from Module b, presented seed-specific expression patterns. *TaPLATZ27* and *TaPLATZ28* exhibited the highest expression at DPA 8, followed by a gradual decrease, subsequently followed by seed development. The expression of *TaPLATZ26*, *TaPLATZ29*, *TaPLATZ30*, *TaPLATZ31*, and *TaPLATZ32* exhibited an up-down-up expression pattern, with a peak at DPA 8, followed by lower expression at DPA 12 and DPA 16, and then an increase again at DPA 20.

### 2.6. Subcellular Localization of TaPLATZ Proteins

**Figure 6:**
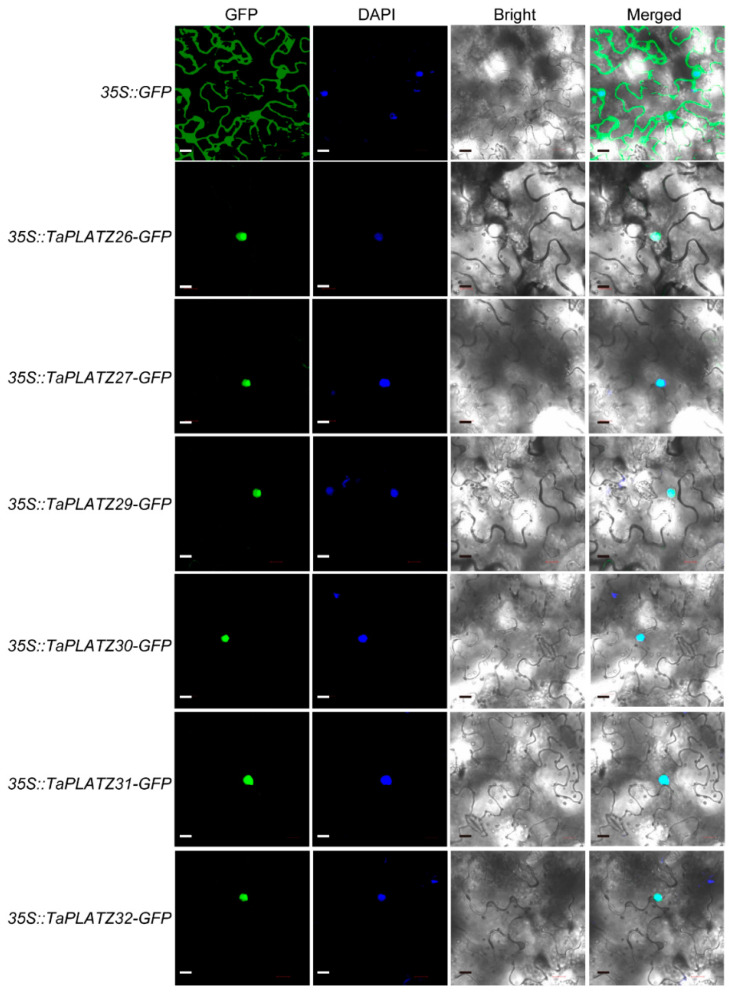
Subcellular localization of TaPLATZ proteins. The localization of the nucleus was detected by DAPI staining. GFP: Green fluorescence indicates the location of TaPLATZ proteins in *N. benthamiana*. DAPI: Blue fluorescence of DAPI indicated the location of the nucleus. Bright light: field of bright light; Merged: merge with the three former images. The scale bar = 20 μm.

The predicted cellular localization by four different software programs showed that six TaPLATZ proteins (TaPLATZ26, TaPLATZ27, TaPLATZ29, TaPLATZ30, TaPLATZ31, and TaPLATZ32) were located in the nucleus (Table S1). Six TaPLATZ proteins were subsequently chosen to verify the subcellular localization by performing the transient expression. They were fused to a green fluorescent protein (GFP), and the constitutive 35S promoter drove all gene cassettes. The free GFP was used as the control. We transiently expressed the resulting constructs in tobacco leaves. Green fluorescence signals of all fusion proteins were localized in the nucleus (Figure 6), consistent with their predicted function as TFs, whereas the control 35S::GFP was detected both in the nucleus and cytoplasm ubiquitously. These results are in accordance with most transcription factors, which were located exclusively at the nucleus, suggesting that the *TaPLATZs* likely perform a function as a conventional transcription factor.

## 3. Discussion

Ancient duplication events and a high rate of retention of extant pairs of duplicate genes have contributed to an abundance of duplicate genes in plant genomes [26,27,28]. A total of 62 *TaPLATZ*s were characterized in wheat, which was almost threefold the numbers of *PLATZs* in Arabidopsis (13), rice (15), and maize (17). The phylogenetic tree of *PLATZ* genes associated with three species revealed that (Figure 1A), the ratio of *PLATZ* ortholog numbers in wheat to rice/maize was as expected—3:1 (wheat: rice and wheat: maize). However, the ratio within some groups was larger than expected. For example, the ratio was about 7:2 or 7:1 in Group II, and the ratio was 4:1 or 4:1 in Group IV. This large divergence suggests that the *PLATZ* gene family might have experienced multiple duplication events during the polyploidization of wheat. Gene duplications are considered to be among the primary driving forces in the evolution of genomes and genetic systems [29]. Duplicate genes provide raw materials for the evolution of mechanism novelties, in turn, facilitate the generation of new functions [29]. While in some of the other cases, the ratio was lower than expected. For instance, wheat orthologs of *ZmPLATZ2* and *ZmPLATZ14* could not be identified in Group III, indicating gene loss during the process of polyploidization of wheat.

Apart from this, among six groups in the phylogenetic tree of three PLATZ families (Figure 1A), genes clustered in Group V and VI have no ortholog gene in neither maize and rice PLATZ family. After sequence alignment, the amino acid sequence of *TaPLATZ*s from Group V and VI showed the highest similarity of *TaPLATZ*s in Group II. The large number of homoeologs can be inferred that most of *TaPLATZ* genes from Group V and VI may be retained after whole-genome duplications (WGDs).

The wheat (BBAADD) has a complex genome consisting of three related sub-genomes that were derived from three different diploid species [30]. The *TaPLATZ*s were unequally distributed in sub-genomes A, B, and D, which included 24, 16, and 20 members, respectively (Table S1, Figure 2). This suggests that homologous genes on the B genome may be absent or become pseudogenes in the lineage leading to wheat [31]. During the evolutionary process of wheat, rapid alterations and sporadic changes in wheat genome took place due to hybridization, polyploidization, domestication, and mutation, resulting in some modifications and a high level of gene loss [32]. Previous reports have stated that the preferential retention of dosage-sensitive genes (e.g., regulatory genes such as transcription factors) and gene loss following WGDs played a significant role in the evolution of eukaryotes [33].

Through the analysis of the evolutionary mechanism, it has been determined that segmental duplication, tandem duplication, and transposition events, including retro-position and replication transposition, had an essential role in the expansion of the number of genes [34,35]. Among these patterns, segmental and tandem duplications are involved in the main patterns in plant gene family expansion [36]. The occurrence of segmental duplications in plant species was considered to be associated with plant polyploidization, followed by inter-chromosomal rearrangements [35].

Tandem duplications are identified by multiple members in one family occurring within the same intergenic region or in neighboring intergenic regions, results from unequal crossing-over and led to increasing or decreasing copy numbers in gene families [36]. Duplication analysis revealed that twenty-one *TaPLATZ* genes were identified as tandem duplicates (Figure 2, Table S6), which account for 37.9% of *TaPLATZ* duplicates, whereas segmental duplication genes account for 3.2%, indicating that tandem duplication pattern probably played a pivotal role in the expansion of *TaPLATZ* gene family. *TaPLATZ* genes are located in the subtelomeric region on chromosome leading to a high percentage of duplication events and results in the expansion of groups. Conversely, genes belonging to groups containing smaller members tended to be located close to the centromere of the chromosomes.

A variety of *TaPLATZ* gene expression patterns was shown in the heat map (Figure 5A). *TaPLATZ*s genes in Module a displayed a continuous expression except in the early stage of seed development, and *TaPLATZ*s genes in Module b presented a seed-specific expression pattern. Moreover, root-specific expression patterns can be found in Module d, indicating the functionally important and nonredundancy of *TaPLATZ* genes. *TaPLATZ* genes clustered in one subfamily could exhibit different expression patterns. For example, members in Group II were clustered into three modules, revealing that functional divergence and the biased expression of duplicated genes appear to be major factors promoting their retention in the genome [37,38]. It is noteworthy that the expression patterns of seven *TaPLATZs* (*TaPLATZ26* to *TaPLATZ32*) contained in Group II were highly similar to those of *ZmPLATZ12* (Fl3) and LOC_Os01g33350, LOC_Os01g33370, which displayed invariant seed-specific expression pattern in the early stage of seeds. These conserved expression patterns reflect that these *TaPLATZ*s may be involved in seed development and maturation, indicating the possibility of their participation in the common metabolic and or developmental processes of wheat [8,12].

Plants can respond and coordinate growth and stress tolerance to promote survival from abiotic and biotic stresses by modifying the production, distribution, or signal transduction of hormones. Zinc-finger transcription factors are a relatively large family of plant transcription factors (approximately 15% of the total), which regulate the expression of several genes in response to abiotic stress such as low temperature, salt, drought, osmotic stress, and oxidative stress [2,39]. AtPLATZ1 and AtPLATZ12 were identified as major nodes to positively regulate the acquisition of desiccation tolerance in Arabidopsis seeds and vegetative tissues [40]. The transcription level of *GhPLATZ1* (*Gossypium hirsutum* PLATZ1) was induced by abiotic and hormone stimuli in 20-day-old seedlings. Ectopic expression of *GhPLATZ1* in Arabidopsis resulted in enhanced insensitivity to osmotic stresses, ABA, and PAC [41]. Similarly, the RNA expression level of *GmPLATZ1* (*Glycine max* PLATZ1) dramatically increased when responding with exogenous ABA application on soybean plants, the leaves mRNA level of *GmPLATZ1* steadily increased after dealing with 24 h drought stress. Furthermore, ectopic expression of *GmPLATZ1* in Arabidopsis showed retarded germination during the early germination process with the addition of mannitol, ABA, and osmotic stress [7]. It is essential to investigate the RNA expression level of *TaPLATZs* under various abiotic stresses in further study, which will facilitate the identification of potential components to coordinate seedling growth during germination.

## 4. Materials and Methods

### 4.1. Plant Growth Conditions

The wheat landrace Chinese spring was planted in plant growth chambers in a 16-h-light/8-h-dark photoperiod at 20 °C in 2019. Tobacco (*Nicotiana benthamiana*) was grown in the plant growth chamber under a 16-h-light/8-h-dark photoperiod at a temperature of 20/25 °C.

### 4.2. Identification of TaPLATZ Family Members in the Wheat

The wheat protein sequence was obtained from the IWGSC database (https://urgi.versailles.inra.fr/download/iwgsc/IWGSC_RefSeq_Annotations/v1.1/). The Hidden Markov Model (HMM) analysis was carried out for the desired sequences search. The HMM profile of the PLATZ (PF04640) downloaded from the Pfam database (http://pfam.xfam.org/) was applied as a query using HMM search program (http://hmmer.janelia.org/) with an E-value cutoff of 1.0. The protein sequences containing complete or partial PLATZ domain, which may be pseudogenes, incomplete assemblies, sequencing errors, or mispredictions [42], were considered as putative *TaPLATZ*s. To eliminate the *TaPLATZ* sequences contained incomplete PLATZ domains, a BLASTP program was performed by using identified amino acid sequences as queries with an *e*-value ≤1 × 10^−3^. Additionally, the amino acid sequences of maize and rice *PLATZ* genes were obtained from PlantTFDB (http://plntfdb.bio.uni-potsdam.de/v3.0/) and GrassTFDB (http://www.grassius.org/grasstfdb.php) databases, which were also used for blasting against wheat genomic reference in the IWGSC v2.0 database.

*TaPLATZ* protein sequences were reconfirmed by Pfam (http://pfam.xfam.org/), SMART (http://smart.embl-heidelberg.de/), and NCBI-CDD (https://www.ncbi.nlm.nih.gov/cdd/) to identify the conserved PLATZ domain. The sequences lacking the PLATZ domain were excluded. All of the non-redundant and high-confidence genes were named after their chromosomal positions on pseudomolecules. The putative TaPLATZ protein sequences were submitted to CDD (https://www.ncbi.nlm.nih.gov/Structure/bwrpsb/bwrpsb.cgi).

### 4.3. Characterization of TaPLATZ: Conserved Motif, PLATZ Domain and Putative Cis-Acting Elements

Conserved motifs of TaPLATZ protein were identified using the MEME website (http://meme-suite.org/tools/meme) [43] with the following parameters: distribution of motifs, 0 or 1 occurrence per sequence; maximum number of motifs, 12; minimum sites, 6; maximum width 50. Visualized of PLATZ domains on each TaPLATZ proteins were performed using TBtoolssoftware [44] (v1.046, Chen, C., GZ, China).

The 2.0-kb upstream of the transcription start site (−1) of *TaPLATZs* was extracted as a promoter to predict cis-acting elements using the PlantCARE (http://bioinformatics.psb.ugent.be/webtools/plantcare/html/) [45]. Then, statistics derived from hits of various cis-acting elements were constructed and displayed by the diagram. Theoretical pI/MW of TaPLATZs was calculated by the Compute pI/MW tool (http://web.expasy.org/compute_pi/).

### 4.4. Phylogenetic Analysis, Collinear Relationships and Classification of PLATZ Genes in Wheat, Maize and Rice

The amino acid sequences of PLATZ derived from maize and rice (Table S1), together with newly identified *TaPLATZs* were used for phylogenetic analysis. All of the amino acid sequences are first aligned by ClustalW with the default parameters. Subsequently, the Bayesian and Neighbor-joining phylogenetic trees were constructed using MEGA software with a bootstrap test of 1000 times [46](v6.0, Tamura, K., Tokyo, Japan). The *TaPLATZs* were classified into different groups according to the topology of the phylogenetic tree and the classification in maize and rice from previous studies [12]. The collinear relationships of orthologous *PLATZ* genes in wheat, maize and rice were displayed using the Circos program [47] (Krzywinski, M., Vancouver, BC, Canada).

### 4.5. Location of TaPLATZ Genes on the Chromosome; Identification of Duplication Genes

The obtained chromosomal location information of *TaPLATZ* genes was visualized by performing MapInspect software (http://www.softsea.com/download/MapInspect.html) (R. van Berloo, Wageningen, The Netherlands)according to their chromosome locus and the length of each chromosome.

The duplication gene pairs in the *TaPLATZ* family were identified by BLASTP based on the criteria of the previous studies [48,49]: (a) the alignment covered >80% of the longer gene; (b) the aligned region had an identity > 80%.

Tandem duplicated PLATZ genes were defined as two or more adjacent homologous genes located physically on a single chromosome with an intergenic region less than 200 kb [50], while homologous genes with an interval greater than 200 kb, or between different chromosomes, were defined as segmentally duplicated genes [51].

### 4.6. Expression Profiles of TaPLATZ

A total of 25 RNA-seq (transcript) data files with wheat tissues (root, leaf, stem, grain and spike) at different developmental stages of the hexaploidy bread wheat (Chuanmai 32, SHW-L1 and Fielder) were obtained from the local database, the abbreviation of each tissue corresponding to the detailed description in Table S7.

The transcriptional results were sorted based on the phylogeny classification and visualized in a heatmap using the ‘ggplot2’ and ‘heatmap’ R-software (Ihaka R, Auckland, CA, USA)package with a normalization according to an individual gene. The bar graph represents the *TaPLATZ*s expression in tissues. The blocks close to Red representing high expression levels, yellow indicating medium expression levels, and blue refers to almost no expression.

For real-time PCR, tissues including root, stem, the third leaf and seeds were obtained from at least three healthy seedlings and three spikelets after sowing. The roots, stems were isolated from the seedling after one-week after imbibition. The seeds were obtained from spikelets at 2, 3, 6, 8, 10, 12, 16, 20, 24, 28, and 32 days after pollination, respectively. RNA was extracted from wheat tissues using the RNA extraction kit (Magen, Beijing, China, Lot: R4165-02), refering to the manufacturer’s instruction for specific steps, and then digested with RNase-free DNase I. The quantity and concentration of RNA were evaluated by Thermo Scientific™ NanoDrop™. The first-strand cDNA was generated using PrimeScript^TM^ RT Reagent Kit (TaKaRa, Kyoto, Japan, Cat. # RR037B). Primer-BLAST (https://www.ncbi.nlm.nih.gov/tools/primer-blast/index.cgi?LINK_LOC=BlastHome) was used to design gene-specific primers. Wheat house-keeping genes 8 and 34 were used as the reference genes.

Real-time quantitative-PCR (qRT-PCR) was carried out using SYBR^®^ Premix Ex Taq™ II (Tli RNaseH Plus) (TaKaRa, Kyoto, Japan, Cat. # RR820A) following the instructions in the manual with a Bio-Rad CFX96^TM^ real-time PCR detection system (BioRad, Berkeley, CA, USA). For normalization, three reference genes were used as internal control genes: *TaGAPDH*, *Ta.7894.3.A1_at* and *Ta.14126.1.S1_at* [52,53]. Three biological replicates of tissues were applied for qPCR analyses. The relative gene expression levels were calculated using the 2^−^^△△CT^ method [54], and the primers used in qRT-PCR are listed in Table S3.

### 4.7. Subcellular Localization of TaPLATZ

The subcellular localization of TaPLATZ protein was predicted according to the results of CELLO Version 2.5 (http://cello.life.nctu.edu.tw/) [55], Plant-mPLoc (http://www.csbio.sjtu.edu.cn/bioinf/plant-multi/) [56], WoLF PSORT (https://wolfpsort.hgc.jp/) and SoftBerry (http://linux1.softberry.com/all.htm). Six seed-specifically expressed TaPLATZs were selected to verify the subcellular localization prediction. The similarity of homologous genes make application difficult, the full-length coding sequences of TaPLATZs without stop codon was synthesized by the company, subsequently inserted into pCAMBIA1300 plasmid driven by 35S promoter. The resulting vectors were *35S::TaPLATZ26-GFP*,*35S::TaPLATZ29-GFP*, *35S::TaPLATZ32-GFP*, *35S::TaPLATZ27-GFP*, *35S::TaPLATZ30-GFP*, and *35S::TaPLATZ31-GFP*, respectively. Agrobacterium-mediated transient transformation of *Nicotiana benthamiana* plants was conducted to check the subcellular locations of *TaPLATZs*. Agrobacterium tumefaciens strain GV3101(Weidi, Shanghai, China) carrying expression construct were grown in LB media with kanamycin and Rifampicin antibiotics, diluted in 1:6 and grown for 9 h at 28 °C, centrifugated for 4000 rpm 5 min, remove supernatant, pellets were resuspended in infiltration medium (10 mM MgCl_2_, 10 mM MES-KOH, PH 5.7, 200 μM acetosyringone). The OD_600_ was adjusted to 0.6–0.9. The resulting culture was infiltrated into 3-week-old *N. benthamiana* leaves using a 1 mL sterile syringe (no needle). At least three replicates were performed. Subcellular localization was observed after infiltration for 2 days. The GFP fluorescent signal was observed and imaged using a confocal laser scanning microscope (LSM 880, Karl Zeiss, Jena, Germany).

## 5. Conclusions

A total of 62 *PLATZ* genes were identified in the wheat genome database, and were distributed unevenly on 15 chromosomes. Chromosome 6 contained the most *TaPLATZ* genes. According to the phylogenetic tree, *TaPLATZ* genes could be classified into six subfamilies (Group I to VI), Group V and Group VI were without orthologues of other *PLATZ* genes in other species. Depending on the type of cis-acting elements, *TaPLATZ* genes may be regulated by a variety of hormones and environmental factors. Gene duplication events analyses suggested that tandem duplication events played a significant role in the expansion of *TaPLATZ* family. Tissue specificity in different developmental stages can be observed in RNA-seq data and qRT-PCR analysis, suggesting the potential role of *TaPLATZs* in tissue differentiation and seed development. This study provides valuable information for further understanding of the evolutionary mechanism and functional traits of the *PLATZ* genes family in wheat.

## Supplementary Materials

The following are available online: https://www.mdpi.com/1422-0067/21/23/8934/s1.

## Abbreviations

DPA	Days post-anthesis
HAI	Hours after imbibition
PLATZ	plant AT-rich protein and zinc-binding protein
NJ	Neighbor-Joining
TFs	Transcription factors
